# British Attitudes Towards Sexuality in Men and Women with Intellectual Disabilities: A Comparison Between White Westerners and South Asians

**DOI:** 10.1007/s11195-015-9423-7

**Published:** 2015-11-03

**Authors:** Deepak Sankhla, Kate Theodore

**Affiliations:** Department of Psychology, Royal Holloway, University of London, Egham, Surrey TW20 0EX UK

**Keywords:** UK, Intellectual disability, South Asian, White Western, Sexuality, Ethnicity, Culture

## Abstract

Although sexuality is a fundamental aspect of human existence, public attitudes towards the sexuality of people with intellectual disabilities may vary. In particular, different ethnic communities may have different perspectives. These differing perspectives may impact on the opportunities and support available for people with intellectual disabilities to express sexuality within ‘normalized’ life experiences. Despite the South Asian population being one of the largest minority ethnic groups residing within the UK, few studies have aimed to understand how South Asian attitudes towards the sexuality of people with intellectual disabilities may differ from White Western perspectives. This study used an online questionnaire to investigate public attitudes towards the sexuality of people with intellectual disabilities within a UK sample (*n* = 331). Attitudes between people from White Western (*n* = 184) and South Asian backgrounds (*n* = 147) were compared with the use of five scales measuring attitudes towards sexuality. Whilst overall attitudes appeared to be generally positive, South Asian participants were found to have significantly more negative attitudes towards the sexual control and sexual rights of people with intellectual disabilities compared to White Westerners. These differences remained significant even after factors known to influence such attitudes were taken into consideration. These findings implicate the need to develop culturally sensitive interventions to improve knowledge and awareness of sexual needs of people with intellectual disabilities. This paper discusses these implications further, the limitations of the present study and suggested directions for future research.

## Introduction

In its recent edition, the Diagnostic and Statistical Manual of Mental Disorders [1] proposes three classification criteria for a diagnosis of an ‘Intellectual Disability’. The individual must have significantly impaired intellectual functioning, which is defined as an Intelligence Quotient (IQ) of 70 or below, which impacts significantly on the individual’s adaptive functioning with an age of onset within the developmental period (i.e. before 18 years of age).

Over the last few decades, there has been a drive for people with intellectual disabilities to integrate into the community and lead normalized life experiences [2–6]. A right to express sexuality has been argued to be an important part in the process of normalization for people with intellectual disabilities [7]. Research has shown sexual and romantic experiences are important for emotional wellbeing and that sexual interests are not known to depend on one’s intellectual functioning [7, 8]. People with intellectual disabilities have expressed their frustration towards denial of their sexual rights, including a lack of privacy necessary for intimacy [9, 10].

Positive attitudes towards people with intellectual disabilities are important in facilitating their inclusion in society [11]. A number of empirical studies have aimed to investigate public attitudes towards different aspects of sexuality in people with intellectual disabilities. These have found that attitudes towards sexuality in people with intellectual disabilities are generally positive from people within lay public populations as well as care staff at community homes and staff from other public services [12–14]. However, these attitudes are less positive when compared to sexual attitudes towards typically developing adults [12]. Furthermore, attitudes towards specific aspects of sexuality such as people with intellectual disabilities becoming parents have been found to be less accepting [12]. Differences in attitudes towards the sexuality of people with intellectual disabilities have been found between people from different occupations. For example, support workers have been found to be more negative in their attitudes towards people with intellectual disabilities becoming parents compared to leisure industry workers [11]. There also appears to be an effect of age, where older people hold more conservative attitudes [12, 13]. Studies have also found more negative attitudes towards the sexuality of men with intellectual disabilities compared to women. Men with intellectual disabilities have been perceived to be less able to control their sexual behaviors and there appears to be more negative attitudes towards their sexual rights and non-reproductive sexual behavior compared to women [14]. A recent study has indicated that people who are more religious are more likely to have more conservative sexual attitudes, both in general and towards people with intellectual disabilities [15]. Attitudes towards sexuality in people with intellectual disabilities may also vary due to beliefs associated with ethnicity and culture.

The Department of Health within the UK has recognized that ‘the needs of people from minority ethnic communities are often overlooked’ [16]. One of the largest minority ethnic groups in the UK includes the South Asian population, which refers to people originating from India, Pakistan, Bangladesh and Sri Lanka [17]. This ethnic group also includes Indian families that have lived in East Africa for a considerable time [18]. This community forms 4 % of the British population. Compared to the general British population, the South Asian populations are more likely to view pre-marital sex as sinful, are more likely to be married at the time of first sex and are less likely to discuss sex with parents during adolescence [19]. Studies within South Asian countries have also found parents avoided discussing sexuality with their children and such discussions took place with peers [20]. South Asian faiths also view aspects of expressing sexuality such as masturbation as shameful, and women in South Asian cultures are expected to be more restricted in their sexuality compared to men [22–24]. All these studies therefore suggest that people from South Asian backgrounds are more conservative in their attitudes towards sexuality in general. Few studies have specifically aimed to understand South Asian attitudes towards *sexuality* in *people with intellectual disabilities*. These studies have mainly been qualitative.

One case study discussed sexuality issues raised by an arranged marriage of a Bangladeshi girl with an intellectual disability called Amina [24]. Parents and relatives of Amina believed that marriage and having children would allow her to get on with her life and overcome her cognitive difficulties. Amina’s father believed that her intellectual disability was treatable and that she would be able to move away and live with her husband in the future. Both parents were positive about the prospect of married life for Amina and anticipated children from the marriage. Similarly, Summer and Jones reported on the case of Mubaraq, a young man in his twenties with intellectual disabilities [25]. Mubaraq’s family were keen for him to be married and believed that this would secure good standing in their community. The family also felt that Mubaraq’s wife would take on a caring role for Mubaraq. Other studies have also been consistent in documenting how people from South Asian backgrounds view marriage and children as a positive future prospect for people with intellectual disabilities and as something that would provide them with a cure [26–28]. However, studies have not explicitly explored South Asian attitudes towards other aspects of sexuality in people with intellectual disabilities.

Therefore, whilst people from South Asian communities are more conservative in their attitudes towards sexuality in general, it may be hypothesized that aspects such as people with intellectual disabilities becoming parents may not have the same taboos associated with Western attitudes. However, most studies in this area have been qualitative and have obtained information via interviews or clinical material. Current knowledge in this area focuses on South Asian people that are parents of a child with an intellectual disability as oppose to more heterogeneous South Asian populations. Whilst case studies have provided useful insights, conclusions drawn may not generalize to the wider South Asian population. Furthermore, these investigations have not specifically aimed to obtain an understanding of South Asian attitudes towards different aspects of sexuality in people with intellectual disabilities.

The present study therefore aimed to address this gap in the literature. It was hypothesized that people from South Asian backgrounds would have more negative attitudes than White Westerners towards all aspects of the sexuality of people with intellectual disabilities, but more positive attitudes towards people with intellectual disabilities becoming parents. It was also hypothesized, as per previous findings, that men with intellectual disabilities would be perceived as more lacking in self-control of their sexuality than women with intellectual disabilities in both ethnic groups. Factors known to influence attitudes towards sexuality and intellectual disabilities were also accounted for within the present study. These included the demographic variables of age, gender and occupation of participants [11–14]. Recognition of and previous contact with intellectual disabilities were also considered as potential covariates, as previous studies have found these to important factors in influencing attitudes [29, 30]. Lastly, attitudes towards sexuality in the typically developing population was another important covariate. This allowed the variance of attitudes towards sexuality in general and attitudes towards the sexuality in people with intellectual disabilities to be separated.

## Method

### Participants

Following University ethics committee approval, 331 adult (aged 18 years and above) participants living in the UK were recruited. Most were recruited online via social networking websites such as Facebook and advertising within the various associated online forums/groups (men’s groups, South Asian culture/religious groups, various occupational/leisure interest groups).

Six participants were recruited and completed paper versions of the questionnaire at a local town centre in East London. Inclusion criteria for the study required participants to be adults living in the UK that described themselves as belonging to either a White Western or South Asian ethnic group. Participants were asked to confirm this on the consent page. In order to aid recruitment, participants were incentivized via an entry to a prize draw to win a £50 online retail voucher.

A calculation of the required sample was undertaken a priori via a power analysis. This was informed from a recent study which found a medium average effect size of 0.3 within similar mean attitudinal differences between South Asians and White Westerners [28]. This study was the closest in relevance to the current study. Based on this medium effect size a power analysis calculated a required a minimum total sample size of 280. Table 1 below details the breakdown of this sample in terms of n values for each questionnaire version and ethnic group.

**Table 1:** N values and percentages within each questionnaire version and ethnic group

Questionnaire version	White Western *n* (%)	South Asian *n* (%)
Male	98 (29.6)	74 (22.4)
Female	86 (26.0)	73 (22.1)

Demographic details of the total, South Asian and White Western sample are depicted in Table 2.

**Table 2:** Demographics representations of the South Asian (*n* = 147) and White Western (*n* = 184) samples

	White Western	South Asian
	*n* (%)	*n* (%)
Gender		
Male	80 (43.5)	74 (50.3)
Female	103 (56)	69 (46.9)
Total response	183 (99.5)	143 (97.3)
Unknown (missing)	1 (0.5)	4 (2.7)
Education		
No qualifications	1 (0.5)	2 (1.4)
GCSE	15 (8.2)	8 (5.40
A-level	35 (19)	19 (12.9)
Undergraduate	75 (40.8)	57 (38.8)
Postgraduate	58 (31.5)	59 (40.10
Total response	184 (100)	145 (98.6)
Missing	0	2 (1.4)
Occupation		
Health/social care	24 (13)	19 (12.9)
Education	11 (6)	6 (4.1)
Homemaker	7 (3.8)	2 (1.4)
Student	5 (2.7)	12 (8.2)
Other employment	122 (66.3)	86 (58.5)
Retired	4 (2.2)	0
Unemployed	3 (1.6)	5 (3.4)
Total response	176 (95.7)	130 (88.4)
Missing	8 (4.3)	17 (11.6)
Age		
18–24	12 (6.5)	17 (11.6)
25–29	48 (26.1)	48 (32.7)
30–34	60 (32.6)	41 (27.9)
35–39	20 (10.9)	14 (9.5)
40–44	18 (9.8)	10 (6.8)
45–49	11 (6)	6 (4.1)
50–54	7 (3.8)	4 (2.7)
55–59	3 (1.6)	5 (3.4)
60–64	4 (2.2)	1 (0.7)
65–69	1 (0.5)	0
Total response	100	146 (99.3)
Unknown (missing)	0	1 (0.7)

### Measures

Each participant completed either measures asking about the sexuality of males or measures asking about the sexuality of females (questionnaire version was a between-subjects factor). This was in order to prevent fatigue effects from the completing a long questionnaire relating to both males and females separately, and biases of order effects [31]. Furthermore, it prevented participant reactivity and social desirable responses. For example, if given both versions, participants may have attempted to show equality in their attitudes towards males and females.

#### Attitudes Towards Sexuality in the General Population (ASQ-GP)

The ASQ-GP [13] was also used to measure attitudes towards sexual expression in typically developing adult (adults without intellectual disabilities). Cuskelley and Gilmore [13] developed the ASQ-GP specifically for this purpose. It has two scales: Sexual Openness (7 items) and Timing (2 items). Only the 7 items measuring sexual openness where used in the study as this component was reported to have high internal consistency at 0.84 by the authors. The ASQ-GP has two different versions, one referring to each gender (male/female). Participants are required to respond to their level of agreement with use of a 6-point Likert scale. Higher scores on the ASQ-GP indicate more positive attitudes.

#### Attitudes Towards Sexuality of Men and Women with Intellectual Disabilities (ASQ-ID)

The most recent revision ASQ-ID developed by Cuskelley and Gilmore [13] was used in the present study. This appears to be the only validated measure that specifically measures attitudes towards the sexuality of men and women with intellectual disabilities.

The scale also has two versions, comprising of the same questions but each asking about either men or women with intellectual disabilities. Participants are required to respond to their level of agreement with use of a 6-point Likert scale. Each question is scored between 1 and 6, with higher scores indicating more positive/liberal attitudes. The scale has four subscales which include Sexual Rights (13 items), Parenting (7 Items), Non-reproductive sexual behavior (5 items) and Self-Control (3 items). These subscales have been documented to have high test–retest reliability (r = 0.91) and good internal consistency (α > 0.90) [13]. Higher scores on the ASQ-ID indicate more positive attitudes. The only modification made to the scale in the present study, with permission from the authors, was that the term “intellectual disability” was replaced with “learning disability” to correspond with familiar British terminology that is better suited to a UK population participating in the study.

#### Measure of Recognition of a Mild Intellectual Disability

A vignette method of assessing recognition of an intellectual disability was employed in this study. Two vignettes (one male and one female) were developed for use in the study and were modified versions of the intellectual disability vignette developed by Scior and Furnham [24]. Modifications that were made included using names that would familiar to both White and South Asian cultures and also describing impairments in adaptive functioning in a more gender neutral manner (details of vignettes can found in the “Appendix” section).

Following presentation of the vignette, respondents are encouraged to label the symptoms depicted by being asked “what, if anything, do you think is wrong with X?” Their responses are then used to assess recognition if an intellectual disability. Following the recognition question, the vignette is also used to orientate respondents to a series of further questions about their views on intellectual disabilities. For the purposes of quantitative analysis, responses were categorized by two raters as either ‘recognized’ or ‘’not recognized’ and inter-rater reliability was also assessed via the Kappa statistic.

#### Measure of Prior Contact with People with Intellectual Disabilities

To asses prior contact the participant was asked about whether they had ever met someone with an intellectual disability and if so in what capacity. Again, for the purposes of quantitative analysis, responses were categorized by two raters as either ‘yes’ or ‘no’ and inter-rater reliability was also assessed.

### Procedure

After participants had the opportunity to read the information sheet and provide informed consent, they were asked to complete the measures in the following order:

1. Socio-demographic information—participants were firstly asked about their socio-demographic details including their age, gender, and ethnicity, whether they had children, whether they were born in the UK and if not their length of residency so far in the UK, their education level and profession.
2. ASQ-GP
3. Vignette: This was introduced by stating that this section was interested in whether people could recognize symptoms of a particular problem. One of two vignettes were presented to participants (either male or female corresponding to the appropriate gender version of the ASQ-GP and ASQ-ID administered). The rationale for using the same gender vignette was to provide some continuity to the questions being asked throughout the questionnaire and to also orientate participants to the appropriate gender referred to in the ASQ-ID. This was then followed by the recognition question: What, if anything, do you think, if anything is wrong with x?
4. Brief information about intellectual disabilities was then presented before questions about prior contact were asked. This information defining intellectual disabilities was derived from the British Psychological Society guidance [32].
5. The ASQ-ID was the last measure to be completed.
6. In the final two pages, participants were asked to provide their email address if they wanted to be entered into the prize draw, were thanked for their participation and reminded of the researchers’ email addresses should they have any further questions.

## Results

Data was assessed and found to follow a normal distribution. Therefore parametric statistical tests were employed using SPSS.

### Univariate Outliers

A total of 15 univariate outliers were identified in the data, as assessed by inspection of a boxplot for values greater than 1.5 box-lengths from the edge of the box. These univariate outliers included four values from the Sexual Openness Scale, two from the Sexual Rights Scale, four from the Parenting Scale, three from the Non-reproductive Sexual Behavior scale and two from the Self-Control Subscale. These outlier values were from eleven participants, ten of whom were from White Western backgrounds. No other explanation was able to be determined as to why the scores of the identified outliers deviated more than three standard deviations from the mean. Due to the large sample size, it was assumed that these outliers were extreme values that were part of the normal distribution as no skew or kurtosis of the distribution was identified in each of the groups. Therefore, data used in the main analyses was analyzed with these outliers included

### Missing Data

Person-mean imputation with a minimum item threshold (80 %) for each of the five scales was used to compensate for missing items values within these scale items. Therefore, the mean of the available items were computed to produce values for the subscales providing that at least 80 % items within each scale were completed. Missing data for the self-control sub-scale was not handled in this way, as there were only three items and also because the reliability analysis demonstrated large reductions in alpha if any items were removed. Therefore any missing data on the items for the self-control subscale resulted in list-wise deletion.

### Reliability Assessments

Inter-rater consistency was assessed for the rating of the recognition measure (whether an intellectual disability was recognized or not recognized from the vignette). Data rated by two raters on a random selection of 10 % of the sample (*n* = 31) demonstrated that the inter-rater agreement (Cohen’s Kappa) for the two raters was κ = 1.0, indicating full level of agreement between the raters. Therefore, a high level of reliability in ratings for the recognition measure for the rest of the data values was also assumed.

Internal consistency was assessed using Cronbach’s alphas for the items on both the male and female versions of the ASQ-GP and ASQ-ID scales. Responses within the fully completed scales were found to range from acceptable (α = 0.73) to high (α = 0.86) levels of reliability in their internal consistency. All scales except the Self-Control scale showed only marginal changes in the alpha values with the removal of particular items.

### Identification of Covariates

In order to control for known confounding factors during hypotheses testing, the total sample was firstly organized into four data sets depending on which questionnaire version was completed (Male or Female Sexuality) and whether participants were White Western or South Asian. These four data sets were assessed for differences between each other via a number of Chi squared tests of associations. This was undertaken for the variables identified from previous research that have also been found to influence attitudes towards sexuality in the general population and the sexuality in people with learning disabilities. These included the variables of, age, gender, education, occupation and ability to recognize a mild intellectual disability as well as previous contact. Wherever necessary, categories were collapsed in order to meet the minimum cell count for each category (*n* = 5).

Pearson’s Chi Squared tests indicated that frequency counts of occupation categories were significantly different in distribution between the two questionnaire versions and/or between the two ethnic groups [χ^2^(3) = 14.91, *p* = 0.002] as was the recognition of a mild an intellectual disabilities [χ^2^(3) = 16.07, *p* = 0.001]. These were therefore included as covariates when testing the main hypotheses.

No significance differences were found for the following variables: Age [χ^2^(6) = 2.64, *p* = 0.85], Gender [χ^2^(3) = 5.16, *p* = 0.16], Education [χ^2^(3) = 3.41, *p* = 0.33] and previous contact [χ^2^(3) = 5.68, *p* = 0.128]. The effect of having children, although not confirmed by previous research, was also proposed to be a potential covariate influencing attitudes towards sexuality, particularly with regards to parenting rights. However, no differences in frequency counts were so found between the samples in whether participants had children [χ^2^(3) = 1.62, *p* = 0.66].

British South Asians showed significantly lower scores compared to White Westerners on sexual openness towards the sexuality of typically developing men [t(167) = −7.58, *p* < 0.001] and women [t(155) = −4.52, *p* < 0.001]. Therefore, sexual openness was also added as a covariate in the main ANCOVA analyses in order to be confident that any differences in variance was due to attitudes *specifically* towards people with intellectual disabilities.

### Attitudes Towards the Sexuality of Men and Women with Intellectual Disabilities

Overall, participants demonstrated generally positive attitudes towards the sexuality of men and women with intellectual disabilities. This was indicated by the total mean response to all the ASQ-ID items for both the White Western (*M* = 5.02, *SD* = 0.58) and South Asian participants (*M* = 4.51 *SD* = 0.76). This was also consistent with attitudes towards sexuality in typically developing men and women as was evident from the mean response on the ASQ-GP in White Westerns (*M* = 5.33, *SD* = 0.61) and South Asians participants (*M* = 4.70, *SD* = 0.82).

### Recognition

Nearly all participants (98 %) provided an answer to the recognition question that was presented after the vignette. Based on the participants that answered this question, recognition of a mild learning disability was generally low. Consistent with previous findings [28], only about one-fifth of this sample (20.8 %) correctly identified that the vignette was describing a person with an intellectual disability. Within this sample of participants that recognized the problem as an intellectual disability, White Westerners showed higher levels of recognition (69.6 %) compared to South Asians (30.4 %) and this difference was found to be statistically significant [χ^2^(1) = 6.85, *p* < 0.05].

### Prior Contact

Most participants stated that they believed they had been in prior contact with a person with a learning disability (72.8 %) mostly via family (22.7 %), friends (21.1 %) or work colleagues (21.8 %). However, it appeared that many participants attributed this prior contact to be with a person with related conditions who may or may not have a co-morbid intellectual disability (e.g. participants reported they had contact with people with Autistic Spectrum Disorders and specific learning difficulties such as Dyslexia) and this may explain the higher scored rate of prior contact despite the low levels of recognition.

### Main Findings

A hypothesis driven analysis was undertaken. This involved individual ANCOVA’s for each of the four scales that measured attitudes towards the sexuality of men and women with intellectual disabilities. For each of the analyses, each of the four scales were entered as a dependent factor and the dichotomous variables of Ethnicity (South Asian/White Western) and Questionnaire Version (Male/Female) were entered as independent factors. The Sexual Openness scale, Occupation and Recognition variables were added as covariates for the ANCOVA analyses. Post-hoc analyses with individual fisher protected *t* tests were then undertaken to assess for the direction of the hypothesized effects of interest. In order to guard against committing a Type 1 error, a Bonferroni correction was applied for the required significance level for these *t* tests where required.

#### Sexual Rights of People with Intellectual Disabilities

A two-way ANCOVA indicated that the effect of Ethnicity was significant [*F*(1291) = 11.855, *p* = 0.001, *η*^2^ = 0.039] indicating a significant difference between mean Sexual Rights scores between the two ethnic groups after controlling for the variance of Sexual Openness, Occupation and Recognition.

Post-hoc Fisher protected *t* tests with a Bonferroni correction and a mean analysis confirmed that compared to White Westerners, British South Asians had significantly lower scores on attitudes towards sexual rights of both men [t(168) = −5.87, *p* < 0.001] and women [t(155) = −4.35, *p* < 0.001] with intellectual disabilities. This indicates that people from South Asian backgrounds were more conservative in their attitudes compared to White Westerners. Means and standard deviations are depicted in Table 3.

**Table 3:** Sexual rights scores: mean, standard deviation and N values

Ethnicity	Questionnaire version	M	SD	N
White Western	Male	4.94	0.60	98
	Female	5.02	0.57	85
	Total	4.98	0.59	183
South Asian	Male	4.31	0.79	72
	Female	4.58	0.70	72
	Total	4.45	0.75	144
Total	Male	4.67	0.75	170
	Female	4.82	0.67	157
	Total	4.74	0.72	327

#### Non-Reproductive Sexual Behavior in People with Intellectual Disabilities

A two-way ANCOVA indicated that the effect of Ethnicity was not significant [F(1, 287) = 2.094, *p* = 0.149, *η*^2^ = 0.005] after controlling for the variance of Sexual Openness, Occupation and Recognition. This indicated that there were no significant statistical differences between the two ethnic groups in their attitudes towards non-reproductive sexual behavior in men and women with intellectual disabilities. Means and standard deviations are depicted in Table 4. These means indicate a non-significant trend in the data where compared to South Asians, people from White Western backgrounds appear to be more positive in their attitudes towards the non-reproductive behavior of both men and women with intellectual disabilities. This trend, although non-significant, is in the hypothesized direction.

**Table 4:** Non-reproductive sexual behavior scores: mean, standard deviation and N values

Ethnicity	Questionnaire version	M	SD	N
White Western	Male	5.18	0.66	97.00
	Female	5.04	0.76	85.00
	Total	5.11	0.71	182.00
South Asian	Male	4.41	0.99	70.00
	Female	4.56	1.03	71.00
	Total	4.48	1.01	141.00
Total	Male	4.86	0.90	167.00
	Female	4.82	0.92	156.00
	Total	4.84	0.91	323.00

#### Parenting Rights of People with Intellectual Disabilities

A two-way ANCOVA indicated that the effect of Ethnicity was not significant [F(1, 284) = 1.522, *p* = 0.218, *η*^2^ = 0.005] indicating that there was no statistical difference in attitudes towards people with intellectual disabilities becoming parents between the two ethnic groups after controlling for the variance of Sexual Openness, Occupation and Recognition. Means and standard deviations are depicted in Table 5. These means indicate a non-significant trend in the data where compared to South Asians, people from White Western backgrounds appear to be more positive in their attitudes towards both men and women with intellectual disabilities becoming parents. This trend is in the opposite direction to what was hypothesized.

**Table 5:** Parenting attitude scores: mean, standard deviation and N values

Ethnicity	Questionnaire version	M	SD	N
White Western	Male	5.08	0.76	93.00
	Female	5.09	0.83	80.00
	Total	5.08	0.79	173.00
South Asian	Male	4.55	0.96	59.00
	Female	4.85	0.90	61.00
	Total	4.70	0.94	120.00
Total	Male	4.87	0.88	152.00
	Female	4.99	0.87	141.00
	Total	4.93	0.87	293.00

#### Sexual Self-Control in People with Intellectual Disabilities

A two-way ANCOVA found the effect of Ethnicity was significant [F(1, 278) = 7.878, *p* = 0.005, *η*^2^ = 0.028] indicating a statistical differences in mean scores of the Self-control scale between the two ethnic groups after controlling for the variance of Sexual Openness, Occupation and Recognition. The effect of questionnaire version was also significant in this model [F(1, 278) = 6.945, *p* = 0.009, *η*^2^ = 0.024] suggesting there was an overall difference in attitudes towards the self-control of sexuality in men compared to women with intellectual disabilities.

Post-hoc Fisher protected *t* tests with a Bonferroni correction found that attitudes between the self-control of sexuality of men and women with intellectual disabilities did not differ. This was both within the South Asian [t(132) = 2.05, *p* = 0.042] and White Western [(t(177) = 2.00, *p* = 0.047] samples. However, there was a trend of the effects in the hypothesized direction and significance in these *t* tests was reached at the uncorrected alpha level (*p* < 0.05).

Post-hoc Fisher protected *t* tests with a Bonferroni correction also confirmed that compared to White Westerners, people from South Asian backgrounds were significantly more negative in their attitudes towards the self-control of sexuality in both men [t(100.11) = −3.65, *p* < 0.001] and women [t(131.05) = −3.01, *p* = 0.003] with intellectual disabilities. Means and standard deviations are depicted in Table 6.

**Table 6:** Self-control attitude scores: mean, standard deviation and N values

Ethnicity	Questionnaire version	M	SD	N
White Western	Male	4.81	0.79	97.00
	Female	5.05	0.82	82.00
	Total	4.92	0.81	179.00
South Asian	Male	4.20	1.18	64.00
	Female	4.60	1.03	70.00
	Total	4.40	1.20	134.00
Total	Male	4.57	1.01	161.00
	Female	4.84	0.95	152.00
	Total	4.70	0.99	313.00

## Discussion

The present study found that compared to White Westerners, the South Asian ethnic group were significantly more negative in their attitudes towards the sexual rights and sexual control of both men and women with intellectual disabilities. These ethnic group differences in attitudes towards sexual rights and control were evident even when factors known to influence such attitudes were taken into consideration. Similar mean trends on the Non-Reproductive Sexual Behavior and Parenting scale were found, but the differences did not reach statistical significance. As with previous findings, attitudes towards the self-control of sexuality were found to be more negative towards men than women with intellectual disabilities, however this was only statistically significant at the uncorrected alpha level (*p* < 0.05).

These findings have clinical implications. For example, health and social care professionals should consider the potential for greater stigma and negative attitudes towards sexual rights and sexual self-control from communities of South Asian service-users with intellectual disabilities. Addressing sexual issues for South Asian people with intellectual disabilities should be approached via interventions involving education teachers and support workers. This is because sexuality is known to be not openly spoken about in South Asian families, especially between children and parents [21].

No significant differences were found between White Westerners and South Asians in their attitudes towards non-reproductive sexual behavior in both males and females with intellectual disabilities. This suggests that UK South Asians, attitudes towards non-reproductive sexual behavior in people with intellectual disabilities have accommodated towards more Western liberal attitudes. Alternatively, the findings may suggest that White Westerners are more conservative attitudes in this area compared to other areas such as sexual rights, which lead to the lack of significant difference between the groups. These findings are at first difficult to interpret, given that South Asian participants were found to have more conservative attitudes towards the sexual rights in people with intellectual disabilities, we would expect the same for non-reproductive sexual behavior. However, when considering the actual questions asked within the non-reproductive behavior scale, four out of the five questions within this scale referred to masturbation practices and one referred to homosexuality. Other aspects of sexual activity such as protected sexual intercourse were not captured and included as being “non-reproductive sexual behavior” in contrast to the sexual rights scale which asked questions concerning a broader range of sexual practices and issues. This helps clarify the findings and we can conclude that either people from South Asian background were less conservative in their attitudes towards masturbation and homosexuality or the White Western were more conservative in these areas compared to other areas such as sexual rights and self-control.

These findings contribute to the knowledge that South Asian and White Western communities have differences in their attitudes towards sexuality in people with intellectual disabilities and that such differences are dependent on particular aspects of sexuality. Therefore health professionals that are faced with sexual issues should be aware of either less or more conservative views of particular aspects of sexuality within between particular ethnic groups.

Although there appeared to be mean differences between the two ethnic groups with regards to attitudes towards people with intellectual disabilities becoming parents, this difference did not reach significance. The non-significant trend on the Parenting scale was actually in the opposite direction to what was hypothesized as White Westerns had higher mean scores (more positive attitudes) than South Asians. Significance may have been achieved with a larger South Asian sample. This is because the number of South Asians that completed the Parenting scale in the present study fell below that calculated size that was required to detect an effect (*n* < 70). This was for both the male (*n* = 59) and female (*n* = 61) versions of the parenting scale. These trends are contradictory to the direction of the effect that was hypothesized as was predicted on the basis of previous qualitative/clinical case studies that suggested that South Asian families may be more favorable to their family members with intellectual disabilities having children of their own. One way of interpreting these trends is that attitudes towards parenting in intellectual disability may only be more positive within a homogenous population of South Asian parents with a child with an intellectual disability, as has been suggested by previous qualitative studies. However, within the lay population recruited in this study, most of whom were unable to recognize an intellectual disability in the vignette, attitudes towards parenting rights may have been equally conservative in both ethnic groups. The implication of this is that services may need to consider that even if family carers of a child with an intellectual disability are relatively positive in their attitudes towards them becoming a parent, they may still be stigmatized in their communities.

Based on previous studies, it was hypothesized that men with intellectual disabilities would be perceived more negatively than women in their self-control of sexuality in both ethnic groups. The present study did not replicate the finding, although trends in the data supported this hypothesis and the effect was significant at uncorrected alpha levels *p* < 0.05). As there appears to be some evidence of such a trend, an important practical implication from this finding includes the need to challenge possible stereotypes that may exist regarding the ability for men with intellectual disabilities in being able to a control their sexuality. One way of challenging such stereotypes and stigma is to develop interventions that aim to educate people about the ability of men with varying degrees of intellectual disabilities to self-control their sexual desires.

The main strength of the present study is its contribution to the knowledge surrounding attitudes in the lay population towards the sexuality of men and women with intellectual disabilities. The present study was the first to quantitatively investigate differences that may exist in these attitudes between people in the lay population that are from White Western or South Asian backgrounds. The study recruited relatively large sample sizes and used robust statistical procedures during the statistical analysis. This included the use of parametric tests that have greater power and attempted to protect against family-wise error in multiple testing by adopting stringent adjustment of significant levels. The present study also controlled for potential confounding factors by identifying unequally represented variables in the data that were known from previous research to influence attitudes towards sexuality. Such variables were entered as covariates in subsequent ANCOVA analyses. Such procedures have their strengths within statistical methods as a way of reducing error variance and elimination of confounds. Another potential extraneous variable that was addressed in the present study is social desirability of responses. This was likely to be minimized given that the data was collected mostly online and in an anonymized form rather than by face-to-face interviews.

As with any investigation, this research study is not without limitations. The present study has its limits in terms of the effect sizes observed and the operationalization of concepts and the measures. Whilst statistically, significant differences were found in this study at corrected levels between the two ethnic groups within sexual rights and self-control scales, such differences may not represent significant differences in real or practical terms. This is because the effect sizes of differences between the two ethnic groups were small. Small effect sizes, even if statistically different may not represent any significant differences at face value. Therefore, real life difference between people from South Asian backgrounds and White Westerners may not be particularly noticeable. The study findings therefore only represent small differences in sexual attitudes between the two ethnic groups, something that may not be noticeable or apparent in real life situations.

Larger effect sizes between the two ethnic groups may be found in studies that continue to explore different factors related to sexual attitudes. One example of this may include comparing differences in attitudes towards sexual practices within and outside a marriage for people with intellectual disabilities. The ASQ-ID and ASQ-GP measures used in this study are therefore limited in that they did not capture this culturally sensitive issue of whether the sexual behaviors and practices being described were being referred to within a marriage.

The measure used to assess recognition in the present study could also be criticized. Whilst the vignette method used in the present study have been shown to be a reliable method in assessing recognition of a mild intellectual disability [29], it is still questionable whether participants are able to fully conceptualize a mild intellectual disability from reading the vignettes used in this study. This is because some participants stated that they had previous contact with a person with intellectual disabilities when actually they were describing contact with people with more specific learning difficulties such as dyslexia or Autistic Spectrum Disorders. Not all people with such disorders have comorbid intellectual disabilities. Methods of recognition that use pictures and videos may provide more ecologically valid measures of recognition of mild-moderate intellectual disabilities.

The presented study provided a definition of an intellectual disability after the recognition measure was administered. This procedure may in itself have resulted in more positive attitudes as it has been argued that lay people discriminate based only on observable behavior as oppose to diagnostic categories [29]. This further suggests that pictures and videos of a person with an intellectual disability may have been a more valid method of assessing recognition of an intellectual disability and orientating people to their attitudes towards sexuality in people with intellectual disabilities. As the measure of prior contact in this study was also problematic, as described above, this suggests a need to develop more reliable measures in assessing whether a person has had prior contact with intellectual disabilities.

The present study considered the South Asian sample as one homogenous group when in reality this community includes people from a diverse range of historical, religious and cultural backgrounds. The same criticism can be applied to the “White Western” group, as this included a small sample of people from American, Australian or European backgrounds. Further research may wish to investigate differences in attitudes towards sexuality in people with intellectual disabilities between different sub-ethnic groups as well as between other ethnic groups. Investigations may also wish to focus on the influence of religious and cultural affiliation on sexual attitudes. Attitudes towards sexual behaviors both within and outside a marriage may be an important factor worthy of further study within the context of religion and culture.

Studies may also wish to use such methods to ask about attitudes towards different severities of intellectual disabilities as the present study’s findings were only applicable to people with mild intellectual disabilities. Lastly, further research may also explore how attitudes towards sexuality towards people from other stigmatized groups may differ compared towards people with intellectual disabilities. This may include, for example, people with physical impairments and people with mental health problems. This would allow the further development and comparison of conceptual models of stigma within contemporary societies.

## Appendix

### Original Vignette 1: Mild Intellectual Disability (Scior and Furnham, 2011)

James is 22 and lives at home with his parents and younger brother. He found school a struggle and left without qualifications. He has had occasional casual jobs since. When his parents try to encourage him to him to make plans for his future, James has few ideas or expresses ambitions that are well out of his reach. Rather than having him at home doing nothing, his mum has been trying to teach James new skills, such as cooking a meal, but James has struggled to follow her instructions. He opened up a bank account with his parents’ help, but has little idea of budgeting and, unless his parents stop him, will spend all his benefits on comics and DVDs as soon as he receives his money.

### Adapted Vignette 1 for Male Version of the Questionnaire

Dylan is 22 and lives at home with his parents and younger brother. He found school a struggle and left without any qualifications. He has had occasional casual jobs since. When his parents try to encourage him to make plans for his future, Dylan has few ideas or expresses ambitions that are well out of his reach. Rather than having him at home doing nothing his parents have been trying to teach Dylan new skills, so he can help with some tasks in the family business, but he has struggled to follow their instructions. He opened up a bank account with his parents’ help, but has little idea of budgeting and, unless his parents stop him, Dylan will spend all his benefits on comics and DVDs as soon as he receives his money.

### Adapted Vignette 2 for Female Version of the Questionnaire

Sonia is 22 and lives at home with her parents and younger brother. She found school a struggle and left without any qualifications. She has had occasional casual jobs since. When her parents try to encourage her to make plans for her future, Sonia has few ideas or expresses ambitions that are well out of her reach. Rather than having her at home doing nothing her parents have been trying to teach Sonia new skills, so she can help with some tasks in the family business, but she has struggled to follow their instructions. She opened up a bank account with her parents’ help, but has little idea of budgeting and, unless her parents stop her, Sonia will spend all her benefits on comics and DVDs as soon as she receives her money.
